# Proceedings of the 7th Annual United States Army Institute of Surgical Research Summer Internship Program 2019

**DOI:** 10.1186/s12967-020-02393-x

**Published:** 2020-07-08

## I1. The United States Army Institute of Surgical Research Summer Internship Program 2019

### Nancy Park^1,2^, John Wall^2^, Michelle Holik^2^, Lucy Schaffer^2^, Aleksa Jovanovic^3^, Shannon Tetens^3^, Eric Roche^3^, Lei Shi^3^, Robert Christy^2,14^, Randolph Stone II^2^, Jessica R. Baker^4^, Christopher Delavan^2^, Isaac E. Abaasah^2^, Barbara A. Christy^2^, James A Bynum^2^, Andrew P. Cap^2,15,16^, Maryanne C. Herzig^2^, Catherine D. Moses^5,15^, Kevin L. Chang^15,20^, Xiaowu Wu^2,15,16^, Christi L. Salgado^2^, Jeffery D. Keesee^2^, Daniel N. Darlington^2,15,16^, James Bynum^2,15,16^, Christopher Haggerty^6^, Alex Trevino^2^, Bopaiah Cheppudira^2^, Leanne C. Duke^2,7,8^, Matthew B. Burgess^2,8^, Luis A. Rodriguez II^2^, Robin M. Kamucheka^2^, Thomas J. Walters^2^, Arezoo Mohammadipoor^2,8^, Madeleine Ausburn^9^, Claudia Millan^2^, Greg Dion^2^, Todd Silliman^10^, Wen Lien^10^, John Decker^10^, Nina Dasari^9^, Emily Boice^2^, Christina Rettinger^2^, Teresa Burke^2^, Heuy-Ching Wang^2^, Michaela R. Priess^9^, Misty M. Strain^2^, Raina Kumar^11^, Roger Chavez^2^, George Dimitrov^11^, Seshamalini Srinivasan^13^, Aarti Gautam^12^, Alex V. Trevino^2^, Molly Williams^12^, Bopaiah Cheppudira^2^, Rasha Hammamieh^12^, John Clifford^2^, Natasha M. Sosanya^2^, Rachel Li^9,2^, David Luellen^2^, Craig Fenrich^2^, Maria Serio-Melvin^2^, Jose Salinas^2^, Sena Veazey^2^, Micaela E. Saathoff^4^, Belinda I. Gómez^14^, Tiffany C. Heard^14^, Jamila M. Duarte^14^, Joshua S. Little^14^, Jose C. Granados^14^, Michael A. Dubick^14^, David M. Burmeister^14^, Chirantan Sen^2,17^, Corinne D. Nawn^2,18^, August N. Blackburn^19^, Kathy L. Ryan^2^, Megan B. Blackburn^2^, Editors: Lauren Cornell^2^ & Whitney Greene^2^

#### ^1^School of Biology, Georgia Institute of Technology, Atlanta, GA, USA, ^2^US Army Institute of Surgical Research, JBSA Fort Sam Houston, TX, USA, ^3^Smith & Nephew, Plc.,Fort Worth, TX USA, ^4^ Truman State University, Kirksville, MO, USA, ^5^ Trinity University, San Antonio, TX, USA, ^6^Wake Forest University, Winston-Salem, NC, 27109, USA, ^7^ Florida State University, Tallahassee, FL, USA, ^8^ Oak Ridge Institute for Science and Education, Oak Ridge, TN, USA, ^9^University of Texas at Austin, Austin, TX, USA, ^10^US Air Force Dental Research and Consultation Services, JBSA Fort Sam Houston, TX USA, ^11^ Advanced Biomedical Computing Center, Frederick National Laboratory for Cancer Research, Frederick, MD, 21702 USA, ^12^ US Army Center for Environmental Health Research, Fort Detrick, MD, USA, ^13^ The Geneva Foundation, Fort Detrick, MD, 21702, USA, ^14^ Damage Control Resuscitation, US Army Institute of Surgical Research, JBSA Fort Sam Houston, TX USA, ^15^Coagulation and Blood Research, United States Army Institute of Surgical Research, Fort Sam Houston, TX, USA, ^16^Department of Surgery, University of Texas Health Science Center, San Antonio, TX USA, ^17^Mississippi State University, Starkville, MS, USA, ^18^University of Texas-San Antonio, San Antonio, TX, USA, ^19^Blackburn Statistics, LLC, San Antonio, TX, USA, ^20^University of Virginia, Morgantown, WV, USA

##### **Correspondence**: Lauren Cornell (lauren.e.cornell.ctr@mail.mil)

###### *J Transl Med* 2020, **18(Suppl 2)**: I1

**Program Details:** Since 1943, the United States Army Institute of Surgical Research (USAISR) has worked to develop advanced solutions and products for injured warfighters as well as other Department of Defense (DoD) beneficiaries and civilians. Out of all of the research laboratories under the U.S. Army Medical Research and Development Command of the U.S. Army Futures Command, the USAISR is the army’s top institution for military medicinal research. The Institute has optimized combat casualty care through three main missions: (1) improve medical care for wounded service members through goal-directed development, (2) oversee the only Burn Center in the DoD and ensuring state-of-the-art care critical care to injured fighters and DoD beneficiaries, and (3) utilize the Joint Trauma System to assess the efficacy of medical care delivered to patients.

Biomedical technologies developed at the USAISR have saved lives not only on the battlefield during Operation Enduring Freedom in Afghanistan and Operation Iraqi Freedom, but also in civilian populations as well; these products include hemostatic dressings, extracorporeal organ support, tourniquets, and burn resuscitation. Through student enrichment, the USAISR has provided learning opportunities for students interested in biomedical research. The Summer Undergraduate Internship Program was established for accomplished undergraduate students from across the nation to hone their skills of scientific inquiry in the context of translational medicine. Students worked alongside a research scientist mentor across the varying departments within the USAISR to find solutions to challenges in combat medicine.

For the 2019 program, twelve students (Fig. 1) were chosen from an applicant pool of several hundred to work on an individual project for ten weeks, from June 3rd to August 9th, 2019. Research task areas included: hemorrhage control and resuscitation, blood and coagulation, burn injury, comprehensive trauma care, intensive care, pain management, multi organ support technology, sensory trauma, and veterinary support.

**Fig. 1:**
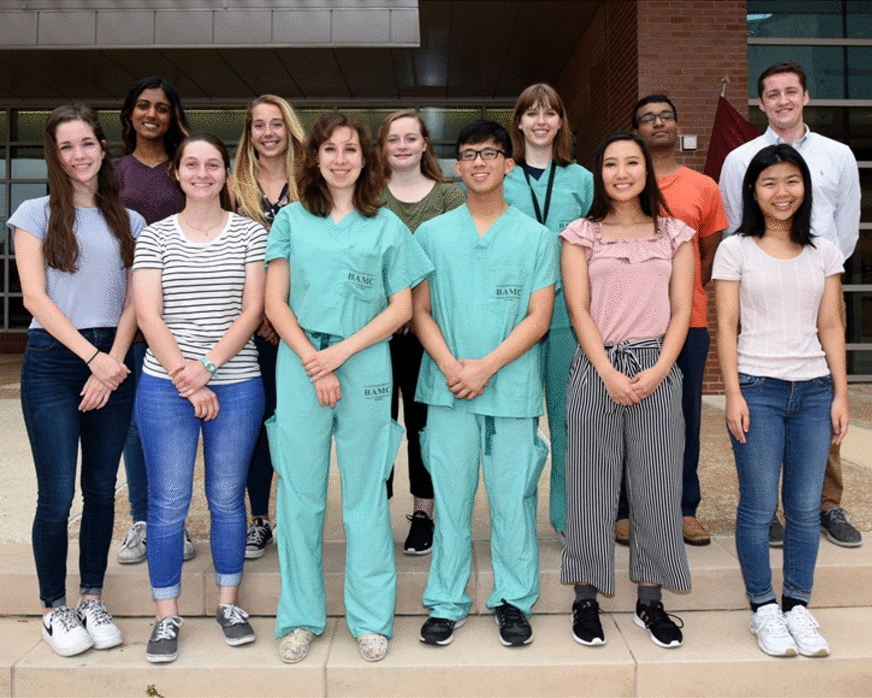
Summer interns outside the USAISR (Bottom row, left to right) Madeleine Ausburn, Michaela Priess, Micaela Saathoff, Kevin Chang, Nancy Park, Rachel Li (Top row, left to right) Nina Dasari, Jessica Baker, Leanne Duke, Catherine Moses, Chirantan Sen, Chris Haggerty. Photo credit: U.S. Army Photo by Dr. Steven Galvan, US Army Institute of Surgical Research Public Affairs

While the primary focus of the program was for students to gain research exposure, students also participated in multiple enrichment activities to gain a better contextual understanding of combat medicine. Interns toured the Center for the Intrepid, attended grand rounds in the USAISR Burn Center Intensive Care Unit, viewed the hyperbaric medicine chamber, presented at weekly journal clubs, and observed a lower negative pressure demonstration. Participants were also invited to attend a local activities. Students learned the rationale behind experimental design, how to interpret and analyze data, and the principles behind scientific communication. Career exploration in research, medicine, and military service were encouraged throughout the duration of the program.

At the end of the internship, students presented posters detailing the results of their projects.

**Eligibility:** In order to be considered for selection to the Summer Undergraduate Internship Program, students must be current U.S. citizens and have completed a year of undergraduate study at an accredited bachelor’s degree program. Because of the nature of the research, applicants enrolled in a Science, Technology, Engineering, or Math (STEM) are preferred.

**Meeting format:** On August 8th, 2019, interns presented the proceedings of their research project to various members of the military scientific community. The USAISR, Naval Medical Research-San Antonio, and the Brook Army Medical Center were invited, and attendees included military personnel, clinicians, postdoctoral researchers, and principal investigators.

**Awards:** After program completion, COL Jerome Buller and Dr. David Burmeister awarded participants with Certificates of Appreciation (Fig. 2).

**Fig. 2:**
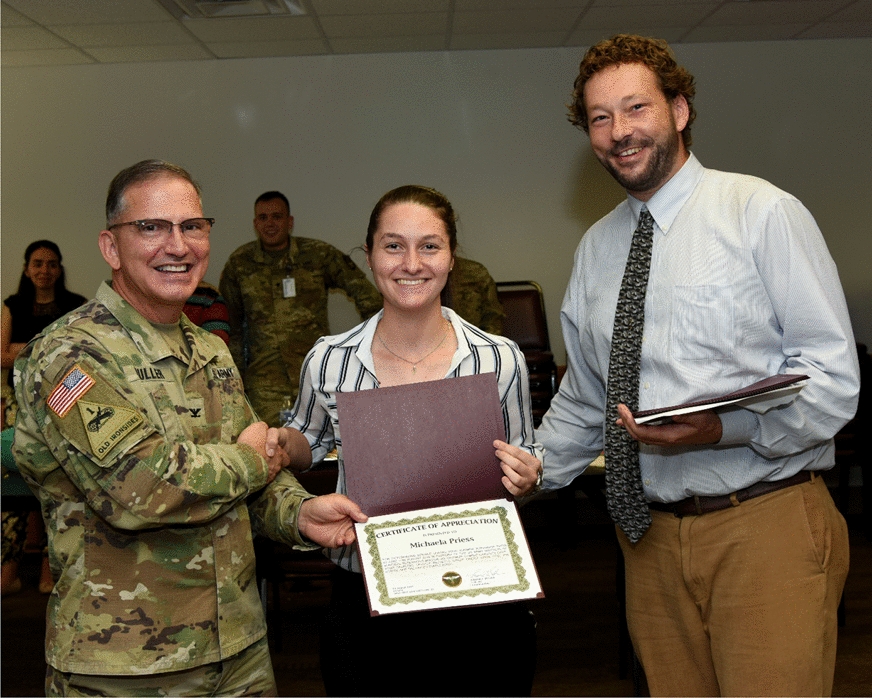
COL Jerome Buller (left) and Dr. David Burmeister (right) presents award to student after completion of internship and poster session. Photo Credit: U.S. Army Photo by Dr. Steven Galvan, US Army Institute of Surgical Research Public Affairs

**Conclusions:** The USAISR Summer Internship Program provides students the opportunity to conduct life-saving research in translational medicine in a state-of-the-art research facility. Participants had the unique experience of conducting research at a military institution, driving innovation specifically for to treat combat casualty. Interns gained laboratory expertise in a variety of fields, such as conducting Western blots, processing animal tissue, and analyzing histological samples. Interns honed their skills of data collection and analysis over the course of the summer on individual research projects. Students also developed proficiency at scientific communication through oral presentations, written abstracts, and poster presentations. Furthermore, participants also observed the meticulous process of experiment development and revision by troubleshooting procedures and consulting field experts. Since 2016, interns have had the opportunity to publish their project abstracts in this journal. Overall, the USAISR presents undergraduate students an immersive research experience to advance their professional goals and allows participants to make a tangible impact on military medicine.

**Acknowledgement:** We express our gratitude to the USAISR Research Directorate for sponsoring the internship program and this publication. We would like to thank Director of Research Dr. Anthony Pusateri, Ms. Melinda Scott, Dr. David Burmeister, and all the principal investigators, as well as the other talented research staff members who committed their time to mentoring students. We would also like to recognize Dr. Steven Galvan for his work in capturing the images for this publication. The research done by the summer interns at USAISR was supported in part by an appointment to the Student Research Participation Program by the Oak Ridge Institute for Science and Education through an interagency agreement between the U.S. Department of Energy and United States Army Medical Research and Development Command (MRDC). We thank our institutional editors, Lauren Cornell, M.S., Ph.D. and Whitney Greene, Ph.D. for founding and editing this student publication program.

**Disclaimers:** The views expressed in this article are those of the authors and do not reflect the official policy or position of the U.S. Army Medical Department, Department of the Army, DoD, or the U.S. Government.

**Funding:** Publication of this article was funded by the USAISR.

**Consent:** The authors have written informed consent from all members in the provided images.

**Application website:** Link to the application website can be found here https://www.orau.org/maryland/.

## P1. Histological analysis of burn wounds after enzymatic debridement

### Nancy Park^1,2^, John Wall^2^, Michelle Holik^2^, Lucy Schaffer^2^, Aleksa Jovanovic^3^, Shannon Tetens^3^, Eric Roche^3^, Lei Shi^3^, Robert Christy^2^, Randolph Stone II^2^

#### ^1^School of Biology, Georgia Institute of Technology, Atlanta, GA, USA, ^2^US Army Institute of Surgical Research, JBSA Fort Sam Houston, TX, USA, ^3^Smith & Nephew, Plc., Fort Worth, TX USA

##### **Correspondence:** Randolph Stone II (randolph.stone4.civ@mail.mil)

###### *J Transl Med* 2020, **18(Suppl 2)**: P1

**Background:** Surgical debridement of burn wounds is considered the gold standard of treatment, but may result in over-debridement and worsen aesthetic and functional outcomes [1]. In military settings, surgical debridement is not performed until patients are transported back to the United States, but early debridement promotes healing and graft success [2]. Enzymatic debridement agents can degrade damaged tissue while minimizing damage to healthy tissue. SN514, a novel metalloprotease, is currently in development as a new enzymatic debridement therapy. Our objective was to evaluate the use of SN514 as a safe and effective non-surgical debridement.

**Materials and Methods:** Twenty partial thickness (PT) and twenty full thickness (FT) burn wounds (3 cm diameter, 100^o^ C) were created on the dorsum of anesthetized Yorkshire pigs on day -4 (n = 8). On day 0, treatments (0.5% SN514, 1.0% SN514, Santyl, Vehicle Control (VC), and No Treatment) were applied and wounds were covered with Tegaderm. Daily reapplication occurred on days 1 and 2. Half of the wounds were biopsied on day 3 by harvesting a strip through the middle of the wound to include normal tissue while the other half of the wounds received one final treatment and were harvested on day 4. Samples were paraffin embedded, sectioned at 5 μm, stained with Masson’s trichrome, and imaged. Average normal dermis depth, wound length, dermis remaining, and area of vascular congestion were measured in ImageJ with Masson’s trichrome stains. One-way analysis of variance and Tukey post hoc tests were conducted in R.

**Results:** The percent of dermis remaining in the wound bed was significantly less when comparing 0.5% SN514 to vehicle control after 72 h of treatment (60.5% vs. 76.9% p < 0.05). SN514 treatment on FT wounds also removed more necrotic tissue than the VC at 72 h (0.5% vs. VC: 39.9% vs. 60.5% p < 0.05; while 1.0% vs. VC: 38.8% vs. 60.5% p < 0.05). After 96 h, only 1.0% SN514 on FT wounds resulted in significant debridement compared to the vehicle control and no treatment samples (1.0% vs. VC: 50.5% vs. 67.0% p < 0.05; while 1.0% vs. NT: 50.5% vs. 68.6% p < 0.05). Less overall debridement was observed after 96 h for all treatments. No punctate bleeding or dermatitis was observed on skin where SN514 was applied.

**Conclusions:** SN514 provided safe debridement in a porcine burn wound model. However, SN514 did not provide complete debridement, as moderately injured tissue remained in the wound bed while more severe collagen denaturation was more conducive to enzymatic debridement. Future work will include testing a reformulation of SN514 and determining if the therapy improves wound healing.

**References**

1. Mosier MJ, Gibran NS. Surgical excision of the burn wound. Clin Plast Surg. 2009;36(4):617-25. 10.1016/j.cps.2009.05.006. PubMed PMID: 19793556.
2. Jaskille A, Shupp J, Jordan M, Jeng J. Critical review of burn depth assessment techniques: Part I. Historical review. Journal of burn care & research: official publication of the American Burn Association. 2009;30(6):937-47.

## P2. Capability of bone marrow, adipose, and umbilical cord MSCs in angiogenesis

### Jessica R. Baker^1^, Christopher Delavan^2^, Isaac E. Abaasah^2^, Barbara A. Christy^2^, James A Bynum^2^, Andrew P. Cap^2^ and Maryanne C. Herzig^2^

#### ^1^Truman State University, Kirksville, MO, USA, ^2^US Army Institute of Surgical Research, JBSA Fort Sam Houston, TX, USA

##### **Correspondence**: Maryanne C. Herzig (maryanne.c.herzig.ctr@mail.mil)

###### *J Transl Med* 2020, **18(Suppl 2)**: P2

**Background:** Angiogenesis is a crucial process in tissue formation and wound healing. Dysfunctional angiogenesis can lead to difficulty healing and repairing dead tissue. Mesenchymal stem cells (MSCs) are stem cells that can differentiate into many cell types [1]. Because they can be derived from medical waste tissue, provide immune-modulatory and anti-inflammatory activity, and are relatively immune-privileged, MSCs show promise as safe allogeneic agents for use in both acute trauma and in wound healing [2]. Here we investigate the ability of human MSCs derived from different tissues to form tubes in vitro under permissive conditions for angiogenesis.

**Materials and Methods:** Human bone marrow, adipose, and umbilical cord MSCs (BM-MSCs, Ad-MSCs, UC-MSCs), human umbilical vein endothelial cells (HUVECs), and cell–matrix (GelTRX) were obtained from commercial sources. Three days prior to the experiment (Day -3) cells were thawed and expanded in separate flasks with appropriate media. MSCs were harvested and plated on matrix in triplicate at 10 k or 5 k cells/well in 24 well plates; HUVEC positive control cells were plated at 24 k or 12.5 k per well. Control wells consisted of cells plated without GelTRX angiogenic matrix. After 4 or 24 h, phase-contrast images were taken using a Leica DNI6000B microscope. Images were analyzed using ImageJ Angiogenesis Analyzer to quantitatively assess tube formation. After imaging, cells were prepared for mRNA isolation and subsequent RT-PCR analysis of angiogenic markers. Cells were also incubated with fluorescently-labeled antibodies specific for human CD31 (PECAM- an angiogenesis protein) as well as phosphatidylserine externalization (an early marker of apoptosis). Fluorescent images were obtained using the Leica microscope.

**Results:** Phase contrast images revealed that MSCs were capable of tube formation under permissive conditions. Angiogenesis was dependent upon cell number and duration. MSC angiogenesis differed from HUVECs; the tubes were slower to form, tube morphology was different, and more numerous but less expansive meshes were formed. Immunofluorescent staining at 24 h revealed that CD31 is prominent in HUVEC tubes yet diffuse for MSCs. At this later point, apoptosis was apparent in both populations as visualized by lactadherin binding to cell surface phosphatidylserine. RT-PCR showed control HUVECs had > 300 fold more CD31 mRNA than control MSCs; both cell types upregulated CD31 upon tube formation.

**Conclusions:** This study confirmed that the angiogenic potential of MSCs may reside not only in their indirect effect on endothelial cells but also on the fact that they themselves were capable of angiogenesis. This potential of MSCs may have implications in wound healing and regenerative medicine in military and civilian trauma.

**References**

1. Chen C, & Liao H, Platelet-rich plasma enhances adipose-derived stem cell-mediated angiogenesis in a mouse ischemic hindlimb model. World J Stem Cells, (2018); (12): 212-227.
2. DeCicco-Skinner K, et al., Endothelial cell tube formation assay for the in vitro study of angiogenesis.J Vis Exp. (2014); (91).

## P3. Evaluation of mitochondrial function of the tissues in rats with polytrauma and hemorrhagic shock

### Catherine D. Moses^1^, Kevin L. Chang^3^, Xiaowu Wu^2^, Christi L. Salgado^2^, Jeffery D. Keesee^2^, Daniel N. Darlington^2^, James Bynum^2^, Andrew P. Cap^2^

#### ^1^Trinity University, San Antonio, TX, USA, ^2^US Army Institute of Surgical Research, JBSA Fort Sam Houston, TX, USA, ^3^University of Virginia, Morgantown, WV, USA

##### **Correspondence**: Xiaowu Wu (xiaowu.wu.civ@mail.mil)

###### *J Transl Med* 2020, **18(Suppl 2)**: P3

**Introduction:** Multiple organ failure (MOF) is a leading cause of post resuscitation death in traumatic injury, which is independent of successful initial resuscitation[1]. Current biomarkers, such as lactate, are not sufficient to evaluate the degree of cellular damage in tissues or to be used as the therapeutic targets to protect organ function following hemorrhagic shock. A close correlation has been demonstrated between mitochondria dysfunction, MOF, and mortality in animal models and clinical observation in various diseases[2], which has not been fully characterized in trauma and hemorrhagic shock. In this study, we hypothesized that the mitochondrial function was significantly decreased in response to acute trauma and hemorrhagic shock.

**Materials and Methods:** Sprague–Dawley rats received control (n = 6) or polytrauma/hemorrhagic shock (TH, n = 4) under isoflurane anesthesia. Polytrauma was induced by: laparotomy, gentle crush of 10 cm-segment of intestine anterior to the cecum, gentle crush of the right and medial liver lobes, bone fracture (right femur), skeletal muscle crush (right hindlimb), and controlled hemorrhage (40% of blood volume) within 30 min after trauma. At 4 h after trauma, the blood sample was collected for liver function test (Alanine Transaminase (ALT) and Aspartate Transaminase (AST) Assay Kit), kidney function test (BUN (Blood Urea Nitrogen) and creatinine (Cr) by iSTAT), and lactate (by iSTAT). The rats were euthanized at 4 h after trauma, and then the liver (non-traumatized), kidney and skeletal muscles (Extensor Digitorum Longus (EDL) and Soleus) were collected to measure mitochondrial function using high-resolution respirometry (OROBOROS Oxygraph 2 K). The oxygen consumption was measured after sequentially adding adenosine-diphosphate (ADP, 5 mM), succinate (10 mM), oligomycin (7.5 µM), carbonylcyanide 4-(trifluoromethoxy) phenylhydrazone (FCCP, 2.5 µM), rotenone (2.5 µM), and antimycin A (12.5 µM) for assessing the activity of mitochondrial complex I, complex II, maximum oxidative phosphorylation (OSPHOS), maximum capacity of electron transport system (ETS), and residual oxygen consumption (ROX) respectively. Data is expressed as mean ± standard error of mean and analyzed using student’s *t* test, significance was set at P < 0.05.

**Results:** Polytrauma/hemorrhagic shock led to a reduction in liver and kidney function (rise in ALT, AST, BUN and Cr), and an elevation in lactate. In parallel with organ dysfunction identified by the functional tests, there was a significant reduction in mitochondrial function of liver and kidney as measured by a significant decline in OSPHOS, complex II activity, and ETS.. Additionally, the mitochondrial function was restored in the soleus but not EDL, suggesting that trauma/hemorrhagic shock induced mitochondrial dysfunction is at least associated with the tissue differential in mitochondria content (Table 1).

**Table 1:** Oxygen Consumption Rates of the Tissues measured by OROBOROS

Respiration (pmol/s/mg)	Liver		Kidney		EDL		Soleus	
	Control	TH	Control	TH	Control	TH	Control	TH
Endogenous	9.0 ± 0.9	8.2 ± 0.9	21.3 ± 7.1	11.7 ± 7.0	4.8 ± 3.4	2.2 ± 0.8	2.2 ± 0.9	2.0 ± 0.4
Substrate	13.5 ± 1.5	12.7 ± 1.6	39.7 ± 12.5	19.0 ± 2.2	26.2 ± 14.8	15.4 ± 6.7	23.5 ± 5.6	35.9 ± 16.1
ADP	31.8 ± 5.3	22.1 ± 5.1	70.9 ± 23.0	30.8 ± 2.8*	75.3 ± 40.7	42.5 ± 17.5	74.3 ± 22.6	121.3 ± 21.3^#^
Succinate	64.5 ± 6.9	42.6 ± 5.0*	109.7 ± 39.0	61.0 ± 7.9	98.3 ± 37.7	50.6 ± 14.8	104.2 ± 22.3	152.0 ± 58.6^#^
Olygomycin	29.6 ± 2.0	24.5 ± 2.9	64.4 ± 16.3	43.1 ± 3.3*	33.8 ± 8.6	16.1 ± 1.9*	36.0 ± 3.6	41.8 ± 8.4^#^
FCCP	75.5 ± 9.0	44.3 ± 6.5*	95.1 ± 26.1	57.8 ± 2.2	76.3 ± 13.6	39.6 ± 9.9*	85.7 ± 20.5	117.0 ± 53.7^#^
Ratenone	39.3 ± 5.3	21.8 ± 2.5*	52.7 ± 13.8	31.8 ± 3.7	34.1 ± 8.3	15.6 ± 1.3*	36.6 ± 5.5	47.4 ± 19.0^#^
Antimycin A	6.5 ± 1.7	6.2 ± 1.2	13.1 ± 1.5	9.2 ± 1.0*	5.1 ± 2.8	3.9 ± 1.9	6.9 ± 3.1	5.1 ± 1.5

*: p < 0.05, significant difference between Control and TH

^#^: p < 0.05, significant difference between EDL and Soleus in TH

**Conclusions:** This preliminary study suggests that acute liver and kidney failure after polytrauma/hemorrhagic shock is associated with mitochondria dysfunction, which in particular is due to a reduction in mitochondrial complex II activity and ETS. However, the underlying mechanisms have not been well defined and will be explored in the future. The capability of assessing mitochondrial function in the tissues will improve understanding the pathophysiology of trauma and hemorrhagic shock, which will ultimately be used as a sensitive biomarker for diagnosis and mitochondrial-directed therapy for hemorrhagic shock induced tissue damage.

**References**

1. Minei JP1, Cuschieri J, Sperry J, Moore EE, West MA, Harbrecht BG, O’Keefe GE, Cohen MJ, Moldawer LL, Tompkins RG, Maier RV. The changing pattern and implications of multiple organ failure after blunt injury with hemorrhagic shock. Crit Care Med. 2012 Apr;40(4):1129-35. 10.1097/ccm.0b013e3182376e9f.
2. Thiessen SE, Van den Berghe G, Vanhorebeek I. Mitochondrial and endoplasmic reticulum dysfunction and related defense mechanisms in critical illness-induced multiple organ failure. Biochim Biophys Acta Mol Basis Dis. 2017 Oct;1863(10 Pt B):2534-2545. 10.1016/j.bbadis.2017.02.015.

## P4. Effect of thermal injury and chronic morphine treatment on the spinal nerve growth factor system

### Christopher Haggerty^1^, Alex Trevino^2^, Robert Christy^2^, Bopaiah Cheppudira^2^

#### ^1^Wake Forest University, Winston-Salem, NC, USA, ^2^US Army Institute of Surgical Research, JBSA Fort Sam Houston, TX, USA

##### **Correspondence**: Bopaiah Cheppudira (bopaiah.p.cheppudira.ctr@mail.mil)

###### *J Transl Med* 2020, **18(Suppl 2)**: P4

**Background:** Burn injury causes debilitating pain to both military and civilian patients. Opioid medications have been an efficacious method to control this pain. However, clinical use of these drugs is limited because of the occurrence of analgesic tolerance [1]. Mature nerve growth factor (mNGF) is known to stimulate growth, differentiation, survival, and maintenance of peripheral sensory and sympathetic neurons following injury. Previous studies from our laboratory [2] and others [3] have also demonstrated that the mNGF signaling pathway plays an important role in the development of morphine-induced analgesic tolerance mechanisms. The roles of mNGF and ProNGF during conditions of both burn injury and analgesic tolerance are not fully understood and are the foci of the present study.

**Materials and Methods:** Two groups of adult male Sprague–Dawley rats (saline control and chronic morphine treatment groups, n = 6/group) were used in this study. All rats were anesthetized and thermal injured. Analgesic tolerance was induced in injured rats by subcutaneously administering 10 mg/kg of morphine in 0.5 ml saline twice per day (morning and evening) for five consecutive days. Control animals received saline treatment. Analgesic tolerance was assessed by measuring paw withdrawal latency (PWL) to thermal stimuli. After final behavioral testing on Day 7 post-injury, rats were euthanized and the spinal cord tissue was harvested and frozen. Additionally, trunk blood was collected and plasma was isolated. NGF and ProNGF protein levels were measured using ELISA.

**Results:** Opioid-induced tolerance was demonstrated through a significant decrease in PWL in thermally injured, morphine-treated rats. The combination of thermal injury and chronic morphine treatment significantly increases the mNGF level in the spinal cord compared to rats that received thermal injury alone. Also, the contralateral side of the spinal cord of thermally injured rats with morphine treatment showed a significant increase in mNGF levels compared to the ipsilateral side. There were no significant changes in the ProNGF levels in either the spinal cord or plasma between saline and morphine treated animals.

**Conclusions:** Our data demonstrates that chronic morphine administration induces analgesic tolerance in thermally injured rats. A combination of thermal injury and chronic morphine treatment significantly alters mNGF protein levels at the spinal cord level. ProNGF levels appear to be unchanged following chronic morphine treatment in the spinal cord and blood plasma. The preliminary findings from this study indicate that mNGF may play a role in morphine-induced analgesic tolerance mechanisms.

**References**

1. Hutchinson et al. Exploring the neuroimmunopharmacology of opioids: an integrative review of mechanisms of central immune signaling and their implications for opioid analgesia. Pharmacol Rev. 2011; 63(3): 772-810.
2. Cheppudira et al. Anti-nerve growth factor antibody attenuates chronic morphine treatment-induced tolerance in rat. BMC Anesthesiology. 2016; 16(1):73.
3. Trang et al. Attenuation of opioid analgesic tolerance in p75 neurotrophin receptor null mutant mice. Neurosci Lett. 2009 Feb 13;451(1):69-73.

## P5. The effect of hypoxia on extracellular vesicles from adipose derived stem cells

### Leanne C. Duke^1,2,3^, Matthew B. Burgess^2,3^, Luis A. Rodriguez II^2^, Robin M. Kamucheka^2^, Thomas J. Walters^2^ and Arezoo Mohammadipoor^2,3^

#### ^1^Florida State University, Tallahassee, FL, USA, ^2^US Army Institute of Surgical Research, JBSA Fort Sam Houston, TX, USA, ^3^Oak Ridge Institute for Science and Education, Oak Ridge, TN, USA

##### **Correspondence:** Arezoo Mohammadipoor (arezoo.mohammadipoor.ctr@mail.mil)

###### *J Transl Med* 2020, **18(Suppl 2)**: P5

**Background:** Acute respiratory distress syndrome (ARDS), the most severe form of acute lung injury, remains a risk factor for death in combat casualty care [1]. Mesenchymal stem cells (MSCs) show promise in the clinical treatment of ARDS; however, factors such as storage requirements make this therapy unfeasible on the battlefield [2]. Since the ability of MSCs may be attributed to their secretome, specifically extracellular vesicles (EVs), we examine the possibility of replacing MSCs with EV treatment. Therefore, we aimed to characterize the properties of EVs from adipose derived stem cells (ASCs) in order to develop a cell-free treatment for lung injury. Since ARDS causes hypoxemia, we also studied the effect of hypoxia on ASCs prior to collection of EVs, with the goal to enhance their therapeutic potential.

**Materials and Methods:** To produce conditioned media (CM), confluent cultures of human ASCs were incubated with serum-free medium (SFM) for 24 or 48 h, either under 21% O_2_ or 2% O_2_. EVs were then isolated from CM by ultracentrifugation. Size distribution of EVs was evaluated using nanoparticle tracking analysis (NTA). Flow Cytometry was performed to assess viability of cells and surface markers of the EVs. Protein content of EVs was assessed by Milliplex and normalized to total protein. All experiments, except for surface marker identification, were performed in triplicates.

**Results:** Following SFM culture, the number of ASCs was reduced in all but the 21% O_2_-24 h group when compared to baseline. Interestingly, the percentage of apoptotic cells was only significantly increased in the 21% O_2_-24 h ASCs group. Flow cytometry showed that EVs derived from the 21% O_2_- and 2% O_2_-groups expressed MSC markers, cellular adhesion molecules, glycosphingolipids, immune regulators, and cell signaling mediators co-expressed with exosome-specific markers. However, EVs from the 2% O_2_ group did not express four proteins detected in EVs from the 21% O2 group. The 21% O2 group EVs expressed proteins related to lymphocyte regulation and cell adhesion. NTA showed no change in mean diameter between time points, but 2%-EVs were significantly larger than 21%-EVs. 21%-24 h-EVs contained high amounts of inflammatory cytokines and growth factors while 2%-24 h-EVs contained smaller amounts of cytokines. 48 h incubation did not significantly alter the protein cargo.

**Conclusions:** This study suggests that serum starvation and hypoxia may affect ASC apoptosis and EV size, surface markers, and protein cargo. In addition, 48-hour incubation does not seem to affect the protein content of EVs. Overall, more in vitro and in vivo *studies* are required to determine the optimal conditions for EV production to enhance their therapeutic benefits for lung injury treatment.

**References**

1. Belenkly, S et al. “Acute Respiratory Distress Syndrome in Wartime Military Burns: Application of the Berlin Criteria.” J Trauma Acute Care Surg. 2014; 76(3) 821-827.
2. Mohammadipoor, A et al. “Therapeutic Potential of Products Derived from Mesenchymal Stem/Stromal Cells in Pulmonary Disease.” Respiratory Research. 2018; 19(218) 1-14.

## P6. Soft tissue characterization of craniomaxillofacial defect in a porcine model

### Madeleine Ausburn^1^, Claudia Millan^2^, Greg Dion^2^, Todd Silliman^3^, Wen Lien^3^, John Decker^3^

#### ^1^University of Texas at Austin, Austin, TX, USA, ^2^US Army Institute of Surgical Research, JBSA Fort Sam Houston, TX, USA, ^3^US Air Force Dental Research and Consultation Services, JBSA Fort Sam Houston, TX, USA

##### **Correspondence:** John Decker (john.f.decker6.mil@mail.mil)

###### *J Transl Med* 2020, **18(Suppl 2)**: P6

**Background:** Since the Vietnam conflict, head and neck trauma has become the second most common battlefield injury. Open mandibular fractures have been reported to occur in 76% of craniomaxillofacial (CMF) combat injuries.^1^ Bone regeneration is key to regaining form, function and psychological well-being of the soldier. Soft tissue closure is critical to successful regeneration. ^2, 3^ The primary goal of this study is to characterize the soft tissue of a porcine model for CMF defects. The second goal is to assess closure techniques to minimize the opening of CMF wounds. We hypothesize that improved CMF wound closure techniques and materials result in differential wound strengths that will resist opening forces in the mobile environment of the face and oral cavity.

**Materials and Methods:** Porcine soft tissue samples were removed postmortem and processed for histologic analysis of various regions of the CMF complex. An Instron Electropuls e3000 machine was utilized for mechanical testing. In short, ninety-four samples of the fresh attached gingiva, buccal mucosa, hard palate, and buccal skin were harvested from Hanford or Yucatan swine. Each sample was cut in half and re-approximated utilizing interrupted, continuous interlocking, or horizontal mattress techniques with a 3-0 chromic gut suture, 3-0 vicryl suture, or Vetbond tissue adhesive. To measure tension, the tissue was pulled at a rate of 50 mm/min near the suture line. Samples were categorized by tissue or suture failure, and their load capacities analyzed.

**Results:** As a wound closure technique, Vetbond provided the greatest resistance to maximum and mean load on all tissues except mucosa. The performance under tension of the continuous interlocking, interrupted, and horizontal mattress techniques varied with each tissue type. As a material, Vicryl provided maximum resistance to load in the mucosa because it is a braided material with a non-smooth surface that provides friction and greatest resistance to load. The chromic gut suture provided the greatest resistance on outer cheek skin. Lastly, load distribution also varied among the tissue types with the greatest amount of resistance to load provided by the outer cheek and gingiva.

**Conclusions:** Primary wound closure can be optimized by selecting the proper closure material and technique for each soft tissue of the CMF region, because it may reduce the incidence of wound dehiscence and subsequent infection.

**References**

1. T.A. Lew, J.A. Walker, J.C. Wenke, L.H. Blackbourne, R.G. Hale, Characterization of craniomaxillofacial battle injuries sustained by United States service members in the current conflicts of Iraq and Afghanistan, J Oral Maxillofac Surg 68(1) (2010) 3-7.
2. Badeau A, Lanham S, Osborn M. Management of Complex Facial Lacerations in the Emergency Department. Clin Pract Cases Emerg Med. 2017; 1(3):162-165
3. Modransky P, Welker B, Pickett JP. Management of Facial Injuries. Vet Clin North Am Equine Pract. 1989 Dec; 5(3):665-82.

## P7. Isolation and characterization of extracellular vesicles from human induced pluripotent stem cells and induced pluripotent stem cell-derived retinal pigment epithelium

### Nina Dasari^1^, Emily Boice^2^, Christina Rettinger^2^, Teresa Burke^2^, Heuy-Ching Wang^2^

#### ^1^The University of Texas at Austin, Austin, TX, USA, ^2^US Army Institute of Surgical Research, JBSA Fort Sam Houston, TX, USA

##### **Correspondence:** Heuy-Ching Wang (heuy-ching.h.wang.civ@mail.mil)

###### *J Transl Med* 2020, **18(Suppl 2)**: P7

**Background:** The mechanism of modern warfare has led to an increase in the incidence of ocular injuries. Traumatic ocular injuries sustained on the battlefield can result in severe vision impairment due to retinal damage. Changes to the retina have been reported with neurodegenerative conditions [1]. Current treatments include utilizing human induced pluripotent stem (iPS) cells, but have limited battlefield viability [2]. Extracellular vesicles (EVs) derived from iPS (iPS-EVs) cells offer a solution as they are proving equally therapeutic, but with greater sustainability and combat-specific utility. Although little is known about them, EVs play a role in intercellular communication, and are thus a promising cell free approach to stimulate tissue regeneration [3, 4]. This study focuses on isolating and characterizing EVs produced from iPS cells and iPS cell-derived retinal pigment epithelial (iPS-RPE) cells.

**Materials and Methods:** Conditioned media from cultured iPS cells and iPS-RPE cells were collected, stored at − 80 °C, and thawed at 4 °C overnight. Media was centrifuged at 4 °C at 300×*g* for 10 min and 10,000×*g* for 30 min. Pellet was disposed of after each spin, and resulting supernatant was spun at 100,000×*g* for 90 min. Final pellet was resuspended in filtered PBS. iPS-EV and iPS-RPE-EV were measured via nanoparticle tracking analysis. iPS-EV samples were characterized to detect surface epitopes via flow cytometry using the MACSPlex Exosome Kit.

**Results:** The iPS-EV mean size increased 147–217 nm over days 1–4, mode size increased linearly 87–196 nm, concentration decreased days 1–3, but increased on day 4 (Fig. 1). There were no significant differences in measurements among EVs from different passages of iPS cells. iPS-EVs were positive for exosome specific markers CD81, CD63, and CD9, and other surface antigens. iPS-RPE-EV concentrations were higher than iPS-EV, which could be due to lower starting volumes of conditioned media. iPS-RPE-EV size increased over time. Apical iPS-RPE-EV concentrations were higher than basal iPS-RPE-EV concentrations (Fig. 2).

**Fig. 1:**
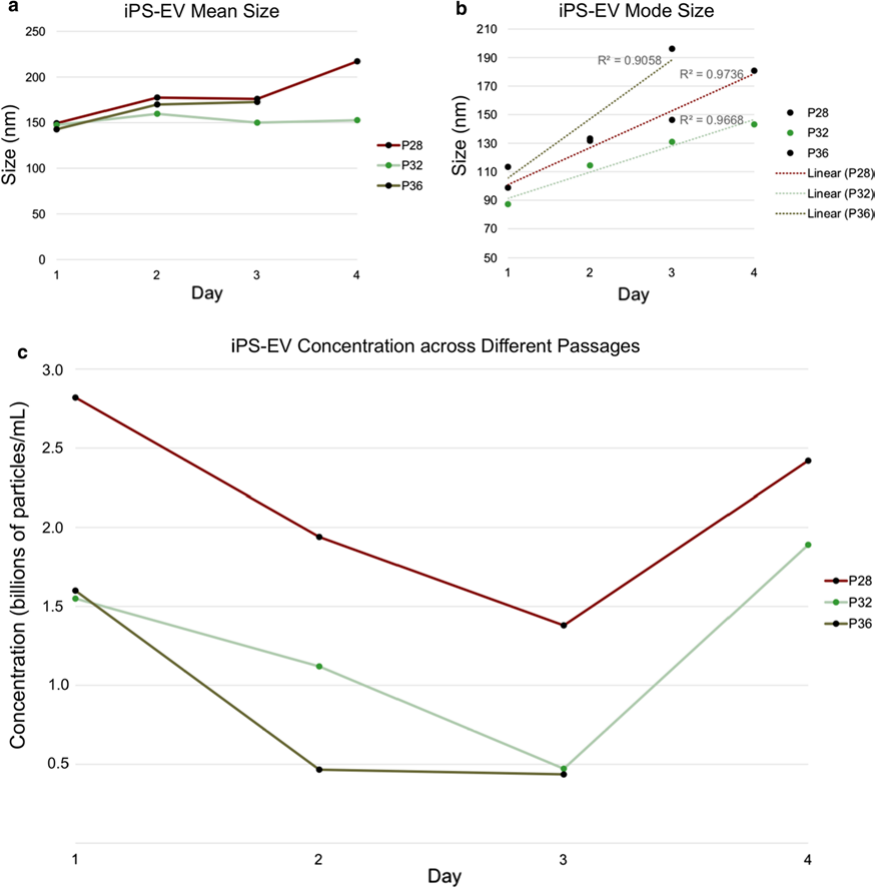
Nanoparticle tracking analysis of iPS-EV A) mean size B) mode size C) concentration by day and passage number

**Fig. 2:**
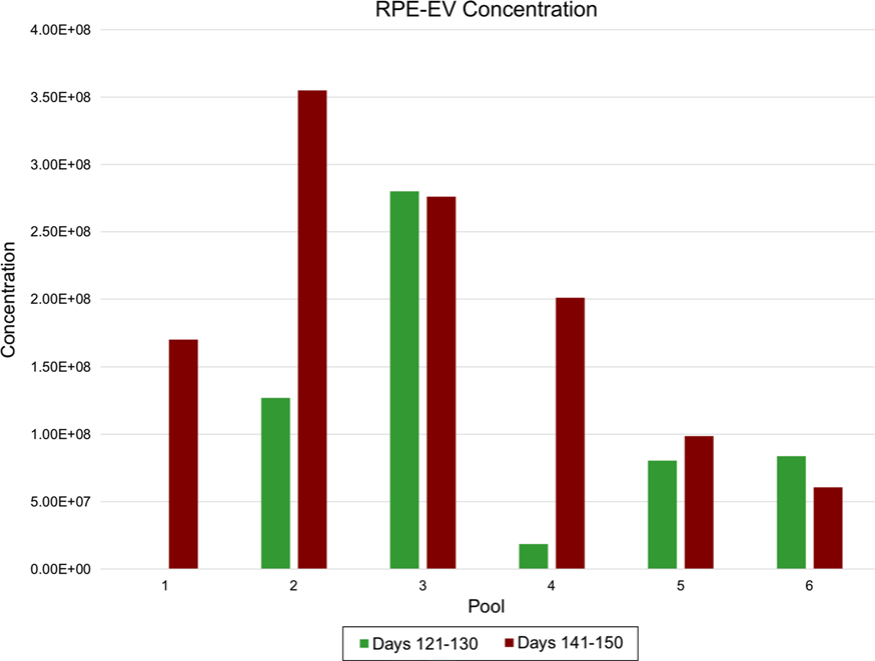
Nanoparticle tracking analysis of iPS-RPE-EV concentration. Pools 1–3 are apical iPS-RPE-EV and pools 4-6 are from basal iPS-RPE-EV

**Conclusions:** The iPS-EV size increased over subsequent days, which correlates to cell growth and proliferation to confluency. Flow cytometry showed iPS-EV contained markers typical of exosomes (CD81, CD63, CD9), and additional surface epitopes characteristic of pluripotent cells. There were no significant differences in concentration. There were no significant differences between different passages of iPS cells. The higher apical iPS-RPE-EV concentrations compared to basal iPS-RPE-EV is consistent with the positioning of RPE cells in the back of the retina and their role in protection and transporting nutrients and ions to the cells nearby (photoreceptors, ganglion cells, etc.).

**References**

1. Childs, C. et al. Investigating Possible Retinal Biomarkers of Head Trauma in Olympic Boxers Using Optical Coherence Tomography. Eye and Brain. 2018; 10:101–110.
2. Chen, F. et al. IPS Cells for Modeling and Treatment of Retinal Diseases. Clin Med. 2014; 3:1511–1541.
3. Laterza, Cecilia, et al. IPSC-Derived Neural Precursors Exert a Neuroprotective Role in Immune-Mediated Demyelination via the Secretion of LIF. Nat Commun. 2013; 4:29.
4. Galieva, Luisa R, et al. Therapeutic Potential of Extracellular Vesicles for the Treatment of Nerve Disorders. Frontiers in Neuroscience. 2019; 13:5.

## P8. Discovering pain biomarkers: Linking differentially-regulated exosomal-miRNAs to target expression in the brain

### Michaela R. Priess^1^, Misty M. Strain^2^, Raina Kumar^3^, Roger Chavez^2^, George Dimitrov^3^, Seshamalini Srinivasan^5^, Aarti Gautam^4^, Alex V. Trevino^2^, Molly Williams^4^, Bopaiah Cheppudira^2^, Rasha Hammamieh^4^, Robert J. Christy^2^, John Clifford^2^, Natasha M. Sosanya^2^

#### ^1^University of Texas at Austin, Austin, TX, USA, ^2^US Army Institute of Surgical Research, JBSA Fort Sam Houston, TX, USA, ^3^Advanced Biomedical Computing Center, Frederick National Laboratory for Cancer Research, Frederick, MD, USA, ^4^US Army Center for Environmental Health Research, Fort Detrick, MD, USA, ^5^The Geneva Foundation, Fort Detrick, MD, USA

##### **Correspondance:** Natasha M. Sosanya (natasha.m.sosanya.ctr@mail.mil)

###### *J Transl Med* 2020, **18(Suppl 2)**: P8

**Background:** Chronic pain is a common issue for approximately 40% of Service Members and 80% of veterans, but little research has been conducted concerning novel pain management treatments. Exosomes are small, endogenous vesicles that participate in intercellular communication by traveling through bodily fluids to deliver cargo, including miRNAs, mRNAs, and proteins to target cells [1]. MAPK14, mTOR, VEGFR2, p70S6K, AKT1 and ERK1/2 are gene targets of miRNA biomarkers we have found to be differentially expressed (DE) in plasma exosomes of rats following spinal nerve ligation (SNL) [2]. Previous research has shown that pathways involving these genes play a role in the development of chronic neuropathic pain, but most studies have only focused on expression of these targets in the spinal cord rather than the brain [3–7]. The objective of this study was to determine whether the targets of DE circulating exosomal miRNAs demonstrate differential expression in specific brain regions following nerve injury.

**Materials and Methods:** Male Sprague–Dawley rats underwent either SNL or sham surgery on the right L5 nerve, under anesthesia [8]. Prior to surgery and on days 3 and 15 post-injury, mechanical allodynia was assessed and plasma was collected. Exosomal RNA was isolated from the plasma, and small RNA was sequenced. DE miRNAs and gene target enrichment for these miRNAs were identified [2]. Brains were harvested from all rats on day 15, and tissue samples were collected from different brain regions. Expression of the gene targets, MAPK14, mTOR, VEGFR2, p70S6K, AKT1, and ERK1/2, and total protein expression in the prefrontal cortex (PFC) was determined by Wes (Protein Simple) and ImageJ analysis.

**Results:** In the left PFC, VEGFR2 and P70S6K (p-Thr421) demonstrated significantly higher expression in the SNL group at day 15. Previously, we have observed that the miRNAs that regulate the pathways of these targets are significantly down-regulated on day 15 [2]. While not significant, MAPK14 demonstrated a sizeable increase in the left PFC of the SNL group. Overall, there were no changes in target expression observed in the right PFC, as expected, because this is the ipsilateral side of injury.

**Conclusions:** Some DE exosomal miRNAs may be affecting expression of their downstream targets in the PFC. Overall, exosomal miRNAs are key regulators, biomarkers, and targets in the treatment of neuropathic pain. Further work still needs to be done to examine target expression of a wider range of DE exosomal miRNAs in different brain regions.

**References**

1. Mcdonald M, Tian Y, Qureshi, et al. Functional significance of macrophage-derived exosomes in inflammation and pain. J Pain. 2014; 155: 1527–1539.
2. Sosanya, N. M., Kumar, R., Clifford, J., et al. Identifying plasma derived extracellular vesicle (EV) contained biomarkers in the development of chronic neuropathic pain. J Pain. 2019; S1526-5900(18)30775-2.
3. Cruz, C. D., and Cruz, F. The ERK 1 and 2 pathway in the nervous system: From basic aspects to possible clinical applications in pain and visceral dysfunction. Curr Neuropharmacol. 2007; 5: 244-252.
4. Um, S. W., Kim, M. J., Leem, J. W., et al. Pain-relieving effects of mTOR inhibitor in the anterior cingulate cortex of neuropathic rats. Mol Neurobiol. 2019; 56: 2482–2494.
5. Ji, R., and Suter, M. R. p38 MAPK, microglial signaling, and neuropathic pain. Mol Pain. 2007; 3.
6. Magnuson, B., Ekim, B., and Fingar, D. C. Regulation and function of ribosomal protein S6 kinase (S6K) within mTOR signaling networks. Biochem. J. 2012; 441: 1–21.
7. Lin, J., Li, G., Den, X., et al. VEGF and its receptor-2 involved in neuropathic pain transmission mediated by P2X_2/3_ receptor of primary sensory neurons. Brain Res Bull. 2010; 83: 284 – 291.
8. Kim, S. H., and Chung, J. M. An experimental model for peripheral neuropathy produced by segmental spinal nerve ligation in the rat. J Pain. 1992; 50: 355–363.

## P9. Applying a user interface and experience approach to an augmented reality-based burn management application: A development and usability study

### Rachel Li^1,2^, David Luellen^2^, Craig Fenrich^2^, Maria Serio-Melvin^2^, Jose Salinas^2^, Sena Veazey^2^

#### ^1^University of Texas, Austin, TX USA, ^2^US Army Institute of Surgical Research, JBSA Fort Sam Houston, TX USA

##### **Correspondence:** Sena Veazey (sena.r.veazey.ctr@mail.mil)

###### *J Transl Med* 2020, **18(Suppl 2)**: P9

**Background:** Burn management is complex process which requires specialized clinical training in order to properly address burn wound size, fluid resuscitation, medication administration, and wound dressing [1, 2]. In order to enhance a non-burn clinician’s ability to comprehensively manage burn patients, we developed a mobile application that utilizes augmented reality (AR). Advantages of an AR headset include hands-free mobility, heads-up display, non-tethered, holographic imagery overlay onto the real environment, and utilization of hand-gestures and voice commands [3]. We re-designed the user interface (UI) to enhance the experience in order to increase its usability and hypothesized that the new UI design will have increased usability.

**Materials and Methods:** We used Visual Studio (Microsoft, Redmond, WA) for software development and Balsamiq Wireframes (Balsamiq, San Francisco, CA) for the UI design and wire-framing. The AR device used was the HoloLens (Microsoft, Redmond, WA). We asked 18 volunteers to participate in a cross matched study to assess the usability of both the old and newly-designed applications. Each volunteer had standardized HoloLens training and was asked to complete 6 standardized tasks for each interface application. Each task was performed on both interfaces. The participants then filled out the SUS surveys and were asked for their overall preference.

**Result:** Mean usability scores (min–max) for the initial and new interface score was 83.75 (62.5–100) and 82.2 (52.5–97.5), respectively. Scores above 68 indicate average usability. Therefore, both the interfaces were indicated to be highly useful. 9 participants preferred the new interface, 8 preferred the old interface, and 1 had no preference.

**Conclusions:** 9 participants (50%) stated that they disliked the pop-up keyboard. A more intuitive and user-friendly Virtual Health application should be developed to address issues with the keyboard of the new interface. An additional study should be conducted to compare the new interface with the initial interface. Based on the usability scores and the participants’ answers, no overall preference between the 2 interfaces was found.

Limitations: The participants required more time with the AR-device for training and familiarity. A notable observation was that the second exposure to one of the applications was generally more favored indicating that familiarity is a factor. Although we implemented a cross match design, users generally took a longer time in familiarizing themselves with the application when first exposed, which may affect perception.

**References**

1. DN, H., *Total Burn Care*, ed. H. DN. 2008, Philadelphia, PA: Elsevier.
2. Kramer GC, L.T., herndon DN, Pathophysiology of burn shock and burn edema, in Total Burn Care. 2002, Elsevier. p. 7–87.
3. Tepper, O.M., et al., Mixed Reality with HoloLens: Where Virtual Reality Meets Augmented Reality in the Operating Room. Plast Reconstr Surg, 2017. **140**(5): p. 1066–1070.
4. Brooke, J., The System Usability Scale. 1986.

## P10. The effect of intravenous xenogeneic adipose-derived stem cells after 40% total body surface area burns in swine

### Micaela E. Saathoff^1^, Belinda I. Gómez^2^, Tiffany C. Heard^2^, Jamila M. Duarte^2^, Joshua S. Little^2^, Jose C. Granados^2^, Robert J. Christy^2^, Michael A. Dubick^2^, David M. Burmeister^2^

#### ^1^Truman State University, Kirksville, MO USA, ^2^Damage Control Resuscitation, US Army Institute of Surgical Research, JBSA Fort Sam Houston, TX USA

##### **Correspondence:** David M. Burmeister (david.m.burmeister3.civ@mail.mil)

###### *J Transl Med* 2020, **18(Suppl 2)**: P10

**Background:** Treatment of severe thermal burn injury (e.g., total body surface area (TBSA) > 20%) often requires wound debridement and intravenous fluid resuscitation with large volumes of lactated Ringer’s [1,2]. While debrided tissue is often discarded as medical waste, it has been found to contain viable adipose-derived stem cells (ASCs) which are easily isolated [3]. Due to their regenerative capabilities, ASCs have been shown to promote wound healing [2,4], but their effects on visceral organs (presumably damaged by systemic inflammation caused by burn injury) are unknown. The goal of this study was to investigate the effects of ASCs on organ function.

**Materials and Methods:** Commercially available ASCs from 3 human donors (RoosterBio) were cultured in 96-well plates. ASC proliferation was monitored after treatment with various resuscitation fluids or with plasma from burned pigs from a previous protocol. A porcine burn model was then used to investigate the effects of ASC infusion on liver and kidney function. Full thickness 40% TBSA contact burns were induced in 18 anesthetized Yorkshire pigs. Animals (n = 6/group) were randomized to 1) no ASCs 2) 5x10^5^ ASCs/kg (low dose), or 3) 5x10^6^ ASCs/kg (high dose). Animals’ drinking fluid also had 5-chloro-2′-deoxyuridine (CldU) (1 mg/mL) to label endogenous proliferating cells. The cells were examined at 24 h in the kidney, liver, and intestine. Cells were also labeled to detect caspase-3 to measure apoptosis.

**Results:***In vitro* results showed that ASC viability, when compared to cell culture media, was significantly increased with pre-burn plasma (p ≤ 0.04) but was significantly decreased by PlasmaLyte (p ≤ 0.005) and fresh frozen plasma (p ≤ 0.003). For in vivo results, two-way ANOVA revealed a significant effect of time for hematological metrics (leukocytes, hematocrit, and total protein), kidney function biomarkers (total urine output, BUN) and liver function biomarkers (total bilirubin, alanine and aspartate aminotransferases). Specifically, biomarkers increased over time, except creatinine (no significance) and bilirubin, which returned to baseline levels by 24 h. Western blot analysis revealed no significant difference between treatment groups for caspase-3 expression in kidney, but higher hepatic caspase-3 in the “low dose” group.

**Conclusions:***In vitro* results demonstrated that cell viability was greatest when treated with pre-burn plasma, suggesting ASC infusion should be prompt following burn. Xenogeneic infusion of ASCs did not improve kidney or liver function, even at large doses. A major limitation of this study was the relatively short duration of treatment. The effect of ASCs on cell proliferation (CldU) is currently under investigation. The ultimate goal of this study is to provide a point-of-care means to ameliorate systemic organ dysfunction caused by severe thermal burn injury.

**References**

1. Gómez B.I. et al., (2018). *Plos One*, 13(5):e0195615
2. Foubert P. et al., (2018). *Burns*, 44(6): 1531–1542
3. Natesan S. et al., (2013). *J Burn Care Res.,* 34(1): 18–30
4. Burmeister D. et al., (2018). *Stem Cells Translational Medicine*, 7(4):360–372

## P11. Regulation of platelet function by adenosine receptors

### Kevin L. Chang^1^, Catherine D. Moses^1^, Daniel N. Darlington^1,2^, Xiaowu Wu^1,2^, James Bynum^1,2^, Andrew P. Cap ^1,2^

#### ^1^Coagulation and Blood Research, United States Army Institute of Surgical Research, Fort Sam Houston, TX, USA, ^2^Department of Surgery, University of Texas Health Science Center, San Antonio, TX USA

##### **Correspondence:** Daniel N. Darlington (daniel.n.darlington.civ@mail.mil)

###### *J Transl Med* 2020, **18(Suppl 2)**: P11

**Background:** Severe trauma and hemorrhage can lead to inhibition of platelet aggregation and elevation in cyclic adenosine monophosphate (cAMP), an effect known to be stimulated by adenosine [1–3]. Because adenosine is released from damaged tissue, it may contribute to the platelet dysfunction seen after severe trauma [1]. Platelets have four G-protein coupled adenosine receptors (A1, A2a, A2b and A3) that have been proposed to stimulate adenylyl cyclase and increase intracellular cAMP [4,5]. Although studies have shown that A2a can inhibit platelet aggregation and elevate cAMP, there is little data showing function of the other receptors.

**Materials and Methods:** Platelet-rich plasma (PRP) was isolated from human whole blood, and centrifuged at 200 g for 10 min. Light transmission aggregometry was performed using a plate reader (Synergy Neo2 Multimode Reader, BioTek) with constant agitation. PRP was stimulated with ADP (100uM) with or without various adenosine agonist and antagonist, including the non-metabolizable adenosine agonist 5-(N-ethyl-carboxamido) adenosine (NECA) in combination with/without antagonists A1 (DPCPX), A2a (Sch 58261), A2b (GS 6201) and A3 (MRS 1220), or with/without agonist A1 (CCPA), A2a (CGS 21680), A2b (Bay 60-6583), A3 (2-Cl-IB-Meca). Cyclic AMP was extracted from 100ul of PRP after adding 1 ml of EtOH, 10 mM ammonium formate, with 10ug/ml cGMP-Br as an internal control. Samples were centrifuged at 20 K g for 10 min, and supernatant dried. Samples were brought up in 200ul of 0.1% formic acid for analysis by Reverse Phase liquid chromatography/Tandem Mass Spectroscopy (Quantiva, ThrermoFisher).

**Results:** We found that adenosine agonist (NECA) inhibits the effects of adenosine diphosphate (ADP)-induced platelet aggregation in a dose dependent manner by elevating cAMP (n = 8). Additionally, A2a receptor antagonist, Sch 58261, was shown to block NECA inhibition of platelet aggregation by 45.7% (n = 9). Following this result, CGS 21680, an A2a receptor agonist, was also shown to inhibit ADP-induced platelet aggregation in a dose dependent manner by elevating cAMP (n = 8). Stimulation of platelets with A2a agonist, but not that of A1, A2b, nor A3, significantly inhibited aggregation and elevated cAMP (n = 8).

**Conclusions:** Adenosine inhibits platelet aggregation in response to natural agonists like ADP through the A2a adenosine receptor by a mechanism that appears to be due to an elevation in intracellular cAMP. These data suggest that the A2a receptor could be potential target for a resuscitation strategy that could attenuate or prevent platelet dysfunction after trauma by preventing synthesis or accelerating removal of cAMP. This study was funded by the USA ISR MRDC.

**References**

1. Darlington DN, Wu X, Keesee JD, AP Cap. Severe Trauma and Hemorrhage Leads to Platelet Dysfunction and Changes in Cyclic Nucleotides In The Rat. Shock. 2019 May in press.
2. Smolenski A. Novel roles of cAMP/cGMP-dependent signaling in platelets. J Thromb Haemost, 2012:10,167–176.
3. Fuentes E, Pereira J, Mezzano D, Alarcon M, Caballero J, Palomo I. Inhibition of Platelet Activation and Thrombus Formation by Adenosine and Inosine: Studies on Their Relative Contribution and Molecular Modeling. PLoS ONE 9(11): e112741.
4. Fuentes E, Fuentes M, Caballero J, Palomo I, Hinz S, El-Tayeb A, Müller CE. Adenosine A2A receptor agonists with potent antiplatelet activity. Platelets. 2018:29, 292–300.
5. Burnstock G. Blood cells: an historical account of the roles of purinergic signaling. Purinergic Signalling 2015:11,411–434.

## P12. Can spectral reflectance differentiate tracheal and esophageal tissue in the presence of bodily fluids?

### Chirantan Sen^1,2^, Corinne D. Nawn^2,3^, August N. Blackburn^4^, Kathy L. Ryan^2^, Megan B. Blackburn^2^

#### ^1^Mississippi State University, Starkville, MS, USA, ^2^US Army Institute of Surgical Research, JBSA Fort Sam Houston, TX, USA, ^3^University of Texas-San Antonio, San Antonio, TX, USA, ^4^Blackburn Statistics, LLC, San Antonio, TX, USA

##### **Correspondence:** Megan B. Blackburn (megan.b.blackburn2.civ@mail.mil)

###### *J Transl Med* 2020, **18(Suppl 2)**: P12

**Background:** Airway compromise is the second leading cause of preventable death in combat fatalities [1]. Endotracheal intubation is a life-saving procedure that frequently fails, leading to patient death through hypoxemia or unrecognized intubation of the esophagus [2]. Current methods of verification of endotracheal tube placement can be subjective and require additional time and manpower [3]. Our previous work [4] confirms a spectral difference between the baseline esophagus and tracheal tissues in both ex vivo and in vivo models. However, no studies have been conducted in the presence of biological fluids, a situation that may occur in patients that have undergone trauma.

**Methods:** The trachea and esophagus were collected from eight pigs used on a previous study. A fiber optic reflection probe, spectrometer, and halogen white light source were used to collect reflectance spectra of tracheal and esophageal tissues. Baseline measurements were taken first without any bodily fluids. Then saline followed by blood and “vomit” (cream of chicken soup) were deposited inside the trachea via syringe. A total of three scans were done in different locations for both the trachea and esophagus in each of the bodily fluids. Using previously identified wavelengths of interest [5], ratios B (561/543) and Y (561/578), termed “detection ratios”, were used to compare the relative intensities of the signals using a likelihood ratio test.

**Results:** Our ex vivo results confirmed previous research [4] of a distinct tracheal spectral profile, with two local minima at 543 nm and 578 nm and one maximum at 561 nm. Importantly, this unique spectral profile was still detectable even with blood, saline and vomit present in the trachea. Ratio B (561/543) was significantly different between trachea and esophagus at baseline and in the presence of all bodily fluids, while ratio Y (561/578) was significantly different at baseline and in the presence of saline and vomit, but not blood (Fig. 1).

**Fig. 1:**
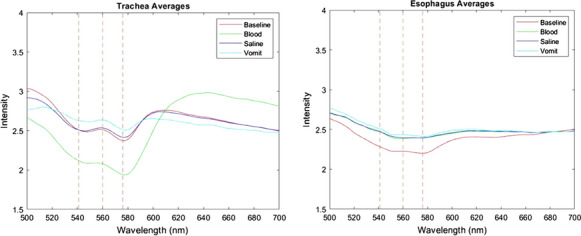
Tracheal and esophageal reflectance spectra at baseline and unaltered with blood, saline or vomit

**Conclusion:** Our data confirm that the trachea has a unique spectral profile. Importantly, the unique tracheal profile, consisting of three distinct points (543, 561, and 578) is still detectable even in the presence of blood, saline, and vomit. Furthermore, the statistical difference in ratios B and Y indicates an ability to distinguish tracheal from esophageal tissue.

**References**

1. Hardy, Garrett B. et al., “Impact of prehospital airway management on combat mortality,” The American Journal of Emergency Medicine. 2018; 36(6): 1032 – 1035.
2. M. A. Cobas et al., “Prehospital intubations and mortality: a level 1 trauma center perspective,” Anesth. Analg. 2009; 109(2): 489–49.
3. P. Rudraraju and L. A. Eisen, “Analytic review: confirmation of endotracheal tube position: a narrative review,” J. Intensive Care Med. 209; 24(5): 283–292.
4. C. D. Nawn et al., “Distinguishing tracheal and esophageal tissues with hyperspectral imaging and fiber-optic sensing,” J. Biomedical Optics. 2016; 21(11): 117004-1–117004-9.
5. Nawn, C.D. et al., “Using spectral reflectance to distinguish between tracheal and esophageal tissue: applications for airway management,” Anesthesia, 2019; 74(3): 340–347.

